# Prevalence and Outcomes of Web-Based Health Information Seeking for Acute Symptoms: Cross-Sectional Study

**DOI:** 10.2196/15148

**Published:** 2020-01-10

**Authors:** Lydia Aoun, Najla Lakkis, Jumana Antoun

**Affiliations:** 1 Department of Family Medicine American University of Beirut Beirut Lebanon; 2 Faculty of Medicine Beirut Arab University Beirut Lebanon

**Keywords:** internet, health information, acute symptoms, acute disease

## Abstract

**Background:**

The literature indicates that Web-based health information seeking is mostly used for seeking information on well-established diseases. However, only a few studies report health information seeking in the absence of a doctor’s visit and in the context of acute symptoms.

**Objective:**

This survey aimed to estimate the prevalence of Web-based health information seeking for acute symptoms and the impact of such information on symptom management and health service utilization.

**Methods:**

This was a cross-sectional study of a convenience sample of 287 Lebanese adults (with a response rate of 18.5% [54/291]) conducted between December 2016 and June 2017. The survey was answered by participants online or through phone-based interviews.

**Results:**

A total of 64.3% of the participants (178/277) reported checking the internet for health information when they had an acute symptom. The rate of those who sought to use Web-based health information first when experiencing acute symptom(s) in the past 12 months was 19.2% (25/130). In addition, 50% (9/18) visited the doctor because of the obtained information, and the rest self-medicated or sought a pharmacist’s advice; the majority (18/24, 75%) improved within 3-4 days.

**Conclusions:**

Higher education level and trust in Web-based medical information were two major predictors of Web-based health information seeking for acute symptoms. Seeking Web-based health information first for acute symptoms is common and may lead to self-management by avoiding a visit to the physician. Physicians should encourage their patients to discuss Web-based health information and guide them toward trusted online websites.

## Introduction

### Background

Since the launch of the World Wide Web in 1990, the number of internet users has increased remarkably (49.6% worldwide [1] and 75.9% in Lebanon [2]) as did the number of Web-based health information seekers [3]. Most internet users visit online websites to find information about physical rather than mental illnesses [4-6] and those mainly related to a diagnosis of a new health problem, an ongoing medical condition, or a prescribed medication [5]. Moreover, most users seek Web-based health information before or after a doctor’s visit [5] to improve their involvement in decision making or supplement information provided by their physicians [7]. They are motivated by the desire for reassurance or a second opinion to challenge other information and to improve their understanding of a condition [8].

Web-based health information seeking is common, and the consumers considered it beneficial. The internet has empowered patients with chronic conditions such as diabetes and cancer [9] and helped patients in making decisions about bariatric surgeries [10] and oncologic management [11]. From the perspective of Web-based health information seekers, the health information they sought was useful [4], had a positive impact on their health [12], and improved their medical information [12] and self-care [7].

### Study Objectives

Most of the literature indicated that Web-based health information seeking is mostly used for well-established diseases. However, only few studies reported health information seeking in the absence of a doctor’s visit [5,7,13,14] and specifically in the context of acute symptoms (symptoms with an abrupt onset and usually a short course, eg, fever, back pain, headache, diarrhea, flu, and urinary frequency). Seeking information on the internet for acute complaints may lead to self-diagnosis and self-medication, which may result in a delay in treatment and incorrect choice of therapies [15,16]. This is more challenging in the context of Web-based health information, as the quality of the information of the websites is not standardized [17]. Therefore, it is worth studying the role of Web-based health information seeking in self-diagnosis of acute symptoms and the consequences of consulting Web-based health information. This survey aimed to estimate the prevalence of Web-based health information seekers for acute symptoms and the impact of such information on symptom management and health service utilization.

## Methods

### Approval

This cross-sectional survey–based study was conducted between December 2015 and June 2016 to identify the proportion of the public who sought to use the internet for information about acute symptoms. Ethical approval was granted by the institution review board of the American University of Beirut.

### Participants and Sample Size

The target population was adults from the general public (aged ≥18 years) residing in Lebanon. The convenience sampling method was selected, and the recruitment method included Google Ads or phone-based interviews. Convenience sampling is widely used in social behavioral studies. As our target population comprised people who used Web-based health information, we elected to use an online recruitment method rather than traditional methods such as posters or inviting people from some geographic areas. In fact, there is growing interest in the literature on the use of Google Ads or Facebook ads to recruit people [18-20].

**Figure 1:**
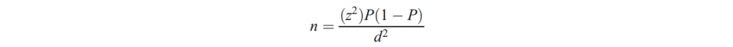
Sample size calculation.

The sample size was calculated with the formula (Figure 1) used in prevalence studies [21,22]. In the absence of the literature reporting this prevalence, we expected the proportion of adults who use the internet for acute symptoms to be similar to that of adults who use the internet for health information, in general. Therefore, we set the expected population proportion as 80% with 95% CIs and the level of precision of estimate within 5%. The sample size required was 246 people.

### Recruitment

**Figure 2:**
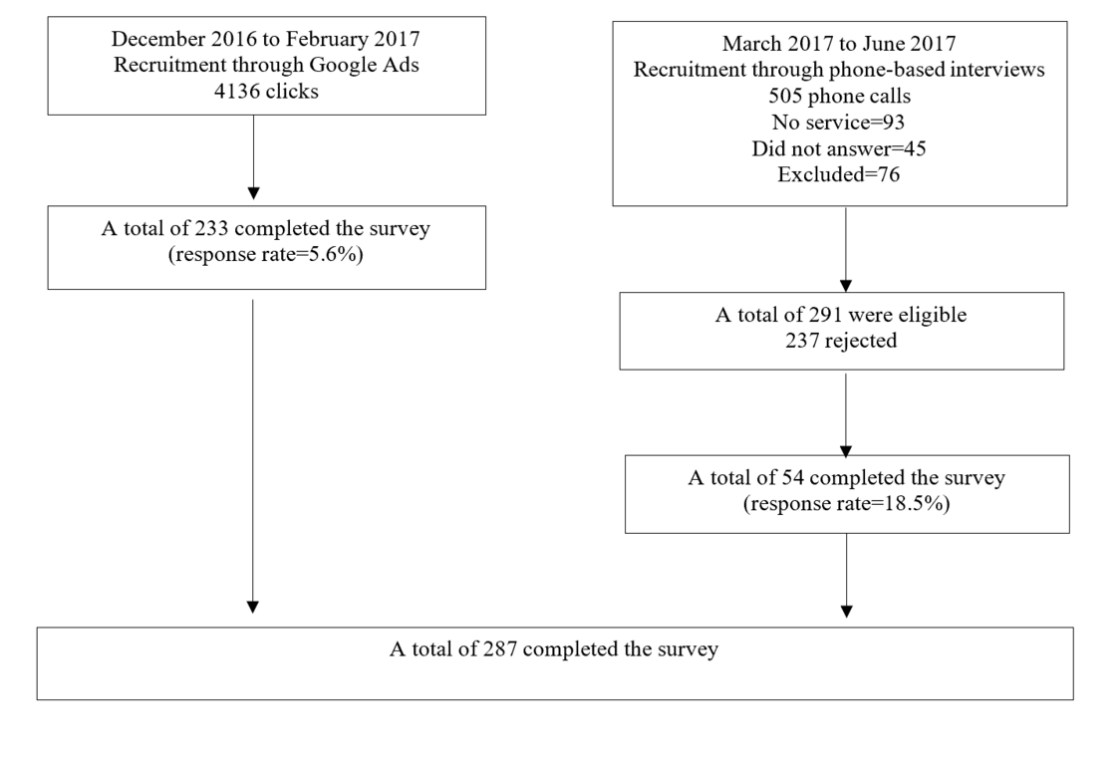
Recruitment of the participants.

A total of 233 participants were first recruited via Google Ads and were asked to complete a Web-based survey. After 3 months, we were not able to recruit enough sample size, so we changed the recruitment to phone-based interviews. The native language of the country is Arabic; however, the country is well known for its trilingual system (Arabic, English, and French). English is taught as a second language in private and public schools and is the language of communication in most universities [23]. For Google Ads, the survey was conducted in English; those who answered the Google Ads were more likely to know English because they were surfing the internet. For the phone-based interview recruitment, the questionnaire was translated into Arabic, the native language of the country, as we may have called people at their households. Using the RAND function in Microsoft Excel, a random list of phone numbers was generated; phone calls were made during fixed times of each day (day and night). Following this, an additional 54 participants were recruited. Figure 2 describes the process of recruiting participants.

### Survey Questions

The survey included information about participants’ demographics, general internet use, and health information seeking. To measure the proportion of participants who sought the internet for information about an acute event, we used the following question: How often do you search the internet for information about an *acute* health event? (An acute illness is defined as a disease with an abrupt onset and usually a short course, eg, headache, fever, flu, pain, diarrhea, urinary frequency, and back pain). Participants were then asked if they had an acute symptom in the past 12 months, whether they sought Web-based health information regarding their acute symptom and their further actions (visit a doctor, pharmacy, or self-medicate), and what were the final illness outcomes (improved or got worse).

### Statistical Analysis

A descriptive analysis was used to describe the demographics, access to Web-based health information, and outcomes. Means were used for continuous variables, and percentages were used for categorical variables. A bivariate analysis using the Chi-square test was used to investigate the relationship between Web-based health information seeking in the acute setting and patients’ actions when they have an acute symptom. The participants’ actions were also correlated with the demographics by using the Chi-square test, except for age, where the correlation was studied using the *t* test/one-way analysis of variance. The statistical significance was set at *P*<.05.

## Results

### Demographics

A total of 287 eligible participants completed the survey. The demographics of the participants are illustrated in Table 1. The mean age of the participants was 46.1 years; 16.7% (N=48) of them were older than 65 years and 6.8% were younger than 20 years. Almost two-thirds of the participants were married (n=139) and had university-level education (n=165), and 77% (n=193) of them had medical coverage. Around two-thirds of the participants did not have a chronic medical illness (n=196) and considered their health to be good (n=122) or very good (n=61).

**Table 1:** Demographics of the participants.

Characteristics		Values
Age (years), mean (SD)		46.1 (19.2)
**Gender (N=242), n (%)^a^**		
	Male	118 (48.8)
	Female	124 (51.2)
**Marital status (N=242), n (%)^a^**		
	Single	82 (33.9)
	Married	139 (57.4)
	Divorced	9 (3.7)
	Widowed	10 (4.1)
	Separated	2 (0.8)
**Educational status (N=240), n (%)^a^**		
	University	165 (57.5)
	Technical training	29 (10.1)
	Secondary school	29 (10.1)
	Primary school	13 (4.5)
	None	4 (1.4)
**Monthly income (US $; N=222), n (%)^a^**		
	<500	43 (15.0)
	500-1300	81 (28.2)
	1300-3300	66 (23.0)
	>3300	32 (11.1)
**Medical coverage (N=252), n (%)^a^**		
	National Social Security Fund	78 (27.2)
	Private insurance	101 (35.2)
	Co-operative	10 (3.5)
	None	59 (20.6)
	Others	4 (1.4)
Chronic medical condition^a^ (N=287), n (%)		91 (31.7)
**Self-perceived health status (N=252), n (%)^a^**		
	Very good	61 (21.3)
	Good	122 (42.5)
	Moderate	52 (18.1)
	Poor	14 (4.9)
	Very poor	3 (1.0)
Have a physician whom they consult on regular basis (N=287), n (%)^a^		148 (51.6)
**Frequency of doctors’ visit in the past 1 year (N=255), n (%)^a^**		
	Never	49 (17.1)
	1-2 times	123 (42.9)
	3 times	32 (11.1)
	≥4 times	51 (17.8)

^a^Missing values.

### Internet Use for Health Information in General and for an Acute Health Event

Participants were provided with the definition of an acute illness: A disease with an abrupt onset and usually a short course, for example, headache, fever, flu, pain, diarrhea, urinary frequency, and back pain. Almost all the participants (235/249, 94.3%) reported general internet use at least twice weekly, whereas one-third of the participants (86/277, 31.0%) reported at least twice weekly internet use for an acute health event specifically (Table 2). One-third of the participants (97/249, 38.9%) rarely or never used the internet for health information, and only 10.3% of them (28/270) rarely or never trusted the health information obtained (Table 2).

Participants who used the internet for an acute illness were likely to be more educated (*P*=.01), have a doctor they regularly consult (*P*=.009), use the internet for health information (*P*≤.001), trust the Web-based health information found (*P*<.001), and have a middle monthly income (*P*=.044). It is also important to note that there was no significant relationship between searching the internet for an acute illness and gender, having a chronic medical condition, perceived health status, frequency of visits to the doctor, or general internet use.

**Table 2:** Internet use for health information in general and for an acute health event.

Characteristics		Value, n (%)
**General internet use**		
	Daily	203 (70.7)
	≥2 times/week	30 (10.5)
	Once weekly	6 (2.1)
	≥2 times/month	1 (0.30)
	Once monthly	13 (4.5)
	Rarely	6 (2.1)
	Never	0 (0)
**Health website use**		
	Daily	39 (13.6)
	≥2 times/week	44 (15.3)
	Once weekly	31 (10.8)
	≥2 times/month	23 (8.0)
	Once monthly	13 (4.5)
	Rarely	68 (23.7)
	Never	28 (9.8)
**Internet search for acute health event^a^**		
	Daily	50 (17.4)
	≥2 times/week	47 (17.3)
	Once weekly	22 (7.7)
	≥2 times/month	41 (14.3)
	Once monthly	13 (4.5)
	Rarely	79 (27.5)
	Never	19 (6.6)
**Trust in Web-based health information obtained**		
	Always	32 (11.1)
	Very often	66 (23.0)
	Sometimes	122 (42.5)
	Rarely	19 (6.6)
	Never	9 (3.1)
	Not applicable	18 (6.3)

^a^An acute health event was defined as an acute illness with an abrupt onset and usually a short course (eg, headache, fever, sore throat, ear pain, cold/flu, diarrhea, urinary symptoms, and acute back pain).

### Description of the Participants Who Had an Acute Symptom in the Past 12 Months

A total of 130 participants (87/130, 45.3%) had an acute symptom in the past 12 months. Their first action was mostly consulting with a physician (52/130, 40.0%) followed by self-medication (45/130, 34.6%), searching the internet for health information (25/130, 19.2%), and seeking a pharmacist’s advice (8/130, 6.2%). Two-thirds of them (85/130, 65.3%) improved in the first 3-4 days after the onset of an acute symptom irrespective of their first action. The most common symptoms were headache, back pain, and diarrhea. Participants who had frequent doctor’s visits, a doctor with whom they consult regularly, or a chronic medical illness were more likely to first seek either Web-based health information or a doctor’s help rather than self-medicate or seek a pharmacist’s advice (Table 3).

Among participants who searched the internet first (n=25), almost half (9/18) consulted a doctor because of the obtained Web-based information; the other half either self-medicated or sought a pharmacist’s advice. Most of them (18/24, 75%) improved in the first 3-4 days.

Among those who self-medicated after searching the internet (n=6), only one participant’s condition did not improve. Similarly, among those who sought care from a doctor after searching the internet (n=8), two participants’ condition did not improve and one participant’s condition worsened. Although many participants (22/25, 88%) found the Web-based information helpful to understand their acute symptoms, two-thirds (15/25, 60%) became anxious, and approximately 72% (18/25) discussed the obtained information with their doctor.

Finally, data analyses were conducted to compare the various demographics and actions of participants among those recruited through Google Ads versus through phone-based interviews. It was shown that participants were similar in both groups, with the exception that those recruited via Google Ads reported higher trust in Web-based health information and were more educated than those recruited via phone-based interviews.

**Table 3:** Bivariate analyses of the first action of the participants with an acute symptom and the various variables.

Variables		First action after an acute symptom				*P* value^a^
		Sought Web-based health information	Sought a doctor’s care	Self-medicated	Sought a pharmacist’s advice	
**Doctors’ visits in the past 12 months (N=121), n (%)**						.002
	0	1 (4.8)	1 (2.0)	11 (26.2)	2 (25.0)	
	1-2	8 (38.1)	15 (30.0)	21 (50.0)	4 (50.0)	
	3	3 (14.3)	10 (20.0)	3 (7.1)	0 (0.0)	
	≥4	9 (42.9)	24 (48.0)	7 (16.7)	2 (25.0)	
**Presence of a chronic medical condition (N=121), n (%)**						.05
	Yes	10 (47.6)	23 (46.0)	10 (23.8)	1 (12.5)	
	No	11 (52.4)	27 (54.0)	32 (76.2)	7 (87.5)	
**Presence of a physician who they consult on a regular basis (N=122), n (%)**						.008
	Yes	16 (76.2)	37 (72.5)	24 (55.8)	1 (14.3)	
	No	5 (23.8)	14 (27.5)	19 (44.2)	6 (85.7)	
**Status of perceived health, n (%)**						.20
	Very good	5 (23.8)	8 (16.0)	11 (25.6)	2 (25.0)	
	Good	10 (47.6)	17 (34.0)	16 (37.2)	3 (37.5)	
	Moderate	5 (23.8)	17 (34.0)	14 (32.6)	0 (0)	
	Poor	1 (4.8)	7 (14.0)	1 (2.3)	3 (37.5)	
	Very poor	0 (0)	1 (2.0)	1 (2.3)	0 (0)	

^a^Chi-square test.

## Discussion

### Principal Findings

Web-based health information seeking is well studied in the context of chronic medical conditions; however, only a few studies analyzed Web-based health information seekers for an acute symptom and the impact of such information on one’s health. This study aimed to add to the literature by examining the prevalence of Web-based health information seeking in the context of an acute illness through a survey. This survey found that many participants (173/277, 62.5%) used the internet for an acute health symptom. Specifically, almost one-fifth (25/130) of those who had an acute symptom in the past 12 months sought Web-based health information first; among those people, two-thirds (9/25, 36.0%) eventually sought care from a doctor based on the information obtained. The majority improved, and the few of them who did not improve or worsened were among those who visited the doctor. The decision to first check Web-based health information was more prevalent among participants who had a doctor whom they consulted regularly, had frequent doctor’s visits in the past 12 months, and had a chronic medical condition.

This study has shown that almost two-thirds (173/277, 62.5%) of the participants used the internet for an acute symptom. This is consistent with the rates of internet usage for general health-related information [3,12,24-26]. Moreover, in comparison with prior studies, Web-based health information seekers are younger [12,25,27,28], highly educated [25,27,28], and consider themselves healthy [27,28]. However, in this study, those who searched the internet for acute symptoms were found to have a middle-level income and not a high income, as seen for general Web-based health information seeking in previous studies [3,25,27,28]. This could be explained by the fact that many participants used Web-based information to self-manage their symptoms. Self-diagnosis and self-medication have been well associated with the socioeconomic status of patients [29-31]. Seeking health information for acute symptoms may have different motives than seeking general health information for chronic or established diseases.

A good number of participants in this survey (25/130, 19.2%) sought Web-based health information first when they had an acute symptom, which is similar to what was found in a study that analyzed the search queries to a patient education orthopedic website: 17% of the patients visited the website for symptoms as compared with 32% for a condition and 22% for a certain treatment [7]. Similarly, 31% of college students sought to use the internet for self-diagnosis [13]. The harms of self-diagnosis through Web-based health information are not well studied. A study among patients with colorectal cancer reported that 25% of the sample consulted the internet before visiting health care providers [14]. Among those who checked information on the internet, 8.2% (n=5) reported that the information influenced their thinking, as it indicated that their condition was not cancer or did not require medical attention. Most of the participants in this study improved after looking up health information on the internet related to their acute symptoms; this might be related to the natural course of most acute symptoms. However, those who did not improve or worsened were among those who visited the doctor after they obtained the information. It is possible that the information obtained was enough for them to decide if they can wait for a natural resolution of the symptoms or there is a need to visit a doctor. In general, the majority of Web-based health information seekers report that this kind of information was helpful and improved their health status or their medical information [4,12,32].

The bivariate analysis found that the decision to search the internet or visit a doctor first was associated with having a doctor with whom they consulted regularly and with a high frequency of doctor’s visits. It seems that participants who did not establish care within the medical system are more likely to self-medicate or visit the pharmacist. Despite that, some participants would seek information on the internet first, and almost half of them who did ended up visiting the doctor. Although physicians remain the most trusted source of medical information [25,33], the literature is mixed about the effect of Web-based information on patients’ confidence in their physicians [34-36]. Discordance between the information obtained from the internet and that from the physician may compromise the trust in the physician [37]. Nevertheless, physicians should encourage their patients to discuss health information obtained from the internet and should guide them toward trusted online websites [38] to help them play an active role in symptom or disease management and control their anxiety.

### Limitations

The generalizability of the data is limited, as the study was conducted among Lebanese adults and had a low response rate. However, the data are a revelation of the magnitude of participants seeking Web-based health information for acute symptoms. Furthermore, two different recruitment methodologies were used sequentially, which lead to more educated participants reporting higher trust in Web-based health information in the Google Ads than in the phone-based interviews. Moreover, there was probably some recall bias, mainly in answering the question addressing health information seeking for the last acute event during the past 12 months.

This survey showed that Web-based health information seeking for acute symptoms is common. However, physicians remain an important source of trustable medical information. Despite seeking Web-based health information first, almost half of the participants eventually visited a doctor and discussed the information that they sought online.

## References

[1] (2017). Internet World Stats.

[2] (2016). Internet World Stats.

[3] Nölke L, Mensing M, Krämer A, Hornberg C (2015). Sociodemographic and health-(care-)related characteristics of online health information seekers: a cross-sectional German study. BMC Public Health.

[4] Reinfeld-Kirkman N, Kalucy E, Roeger L (2010). The relationship between self-reported health status and the increasing likelihood of South Australians seeking Internet health information. Aust N Z J Public Health.

[5] Rice RE (2006). Influences, usage, and outcomes of internet health information searching: multivariate results from the Pew surveys. Int J Med Inform.

[6] Dickerson S, Reinhart AM, Feeley TH, Bidani R, Rich E, Garg VK, Hershey CO (2004). Patient internet use for health information at three urban primary care clinics. J Am Med Inform Assoc.

[7] Shuyler KS, Knight KM (2003). What are patients seeking when they turn to the Internet? Qualitative content analysis of questions asked by visitors to an orthopaedics Web site. J Med Internet Res.

[8] Powell J, Inglis N, Ronnie J, Large S (2011). The characteristics and motivations of online health information seekers: cross-sectional survey and qualitative interview study. J Med Internet Res.

[9] Shaw RJ, Johnson CM (2011). Health information seeking and social media use on the internet among people with diabetes. Online J Public Health Inform.

[10] Paolino L, Genser L, Fritsch S, De' Angelis N, Azoulay D, Lazzati A (2015). The web-surfing bariatic patient: the role of the internet in the decision-making process. Obes Surg.

[11] Shen MJ, Dyson RC, D'Agostino TA, Ostroff JS, Dickler MN, Heerdt AS, Bylund CL (2015). Cancer-related internet information communication between oncologists and patients with breast cancer: a qualitative study. Psychooncology.

[12] Lorence DP, Park H, Fox S (2006). Assessing health consumerism on the web: a demographic profile of information-seeking behaviors. J Med Syst.

[13] Basch CH, MacLean SA, Romero R, Ethan D (2018). Health information seeking behavior among college students. J Community Health.

[14] Thomson MD, Siminoff LA, Longo DR (2012). Internet use for prediagnosis symptom appraisal by colorectal cancer patients. Health Educ Behav.

[15] Bennadi D (2013). Self-medication: A current challenge. J Basic Clin Pharm.

[16] Ruiz ME (2010). Risks of self-medication practices. Curr Drug Saf.

[17] Fahy E, Hardikar R, Fox A, Mackay S (2014). Quality of patient health information on the internet: reviewing a complex and evolving landscape. Australas Med J.

[18] Jones RB, Goldsmith L, Williams CJ, Kamel Boulos MN (2012). Accuracy of geographically targeted internet advertisements on Google AdWords for recruitment in a randomized trial. J Med Internet Res.

[19] Lane TS, Armin J, Gordon JS (2015). Online recruitment methods for web-based and mobile health studies: a review of the literature. J Med Internet Res.

[20] Gross MS, Liu NH, Contreras O, Muñoz RF, Leykin Y (2014). Using Google AdWords for international multilingual recruitment to health research websites. J Med Internet Res.

[21] Arya R, Antonisamy B, Kumar S (2012). Sample size estimation in prevalence studies. Indian J Pediatr.

[22] Lwanga SK, Lemeshow S, World Health Organization (1991). Sample Size Determination in Health Studies: A Practical Manual.

[23] Bacha NN, Bahous R (2011). Foreign language education in Lebanon: a context of cultural and curricular complexities. J Lang Teach Res.

[24] Powell JA, Darvell M, Gray JA (2003). The doctor, the patient and the world-wide web: how the internet is changing healthcare. J R Soc Med.

[25] Hesse BW, Nelson DE, Kreps GL, Croyle RT, Arora NK, Rimer BK, Viswanath K (2005). Trust and sources of health information: the impact of the Internet and its implications for health care providers: findings from the first Health Information National Trends Survey. Arch Intern Med.

[26] Alkhatlan HM, Rahman KF, Aljazzaf BH (2018). Factors affecting seeking health-related information through the internet among patients in Kuwait. Alexandria J Med.

[27] Miller LM, Bell RA (2012). Online health information seeking: the influence of age, information trustworthiness, and search challenges. J Aging Health.

[28] Cotten SR, Gupta SS (2004). Characteristics of online and offline health information seekers and factors that discriminate between them. Soc Sci Med.

[29] Awad A, Eltayeb I, Matowe L, Thalib L (2005). Self-medication with antibiotics and antimalarials in the community of Khartoum State, Sudan. J Pharm Pharm Sci.

[30] Torres N, Chibi B, Middleton L, Solomon V, Mashamba-Thompson T (2019). Evidence of factors influencing self-medication with antibiotics in low and middle-income countries: a systematic scoping review. Public Health.

[31] Alhomoud F, Aljamea Z, Almahasnah R, Alkhalifah K, Basalelah L, Alhomoud FK (2017). Self-medication and self-prescription with antibiotics in the Middle East-do they really happen? A systematic review of the prevalence, possible reasons, and outcomes. Int J Infect Dis.

[32] McMullan M (2006). Patients using the Internet to obtain health information: how this affects the patient-health professional relationship. Patient Educ Couns.

[33] AlGhamdi KM, Moussa NA (2012). Internet use by the public to search for health-related information. Int J Med Inform.

[34] Silver MP (2015). Patient perspectives on online health information and communication with doctors: a qualitative study of patients 50 years old and over. J Med Internet Res.

[35] Tanis M, Hartmann T, Te Poel Fam (2016). Online health anxiety and consultation satisfaction: a quantitative exploratory study on their relations. Patient Educ Couns.

[36] Tan SS, Goonawardene N (2017). Internet health information seeking and the patient-physician relationship: a systematic review. J Med Internet Res.

[37] Lu T, Xu YC, Wallace S (2017). Internet usage and patient's trust in physician during diagnoses: a knowledge power perspective. J Assoc Inf Sci Technol.

[38] Hou J, Shim M (2010). The role of provider-patient communication and trust in online sources in Internet use for health-related activities. J Health Commun.

